# Trenches reduce crop foraging by elephants: Lessons from Kibale National Park, Uganda for elephant conservation in densely settled rural landscapes

**DOI:** 10.1371/journal.pone.0288115

**Published:** 2023-07-26

**Authors:** Allison Rogers, Adrian Treves, Richard Karamagi, Moses Nyakoojo, Lisa Naughton-Treves

**Affiliations:** 1 Nelson Institute for Environmental Studies, University of Wisconsin-Madison, Madison, Wisconsin, United States of America; 2 Kiko Town Council, Kabarole, Uganda; 3 Department of Geography, University of Wisconsin-Madison, Madison, Wisconsin, United States of America; Feroze Gandhi Degree College, INDIA

## Abstract

Crop loss to wildlife, particularly elephants, threatens livelihoods and support for conservation around many protected areas in Africa and Asia. Low-cost elephant barriers have been successfully deployed in savannas but seldom tested around isolated forest parks where the stakes are high for local farmers and isolated elephant populations. We measured the effectiveness of a series of ≥3 m deep trenches dug by farmers neighboring Kibale National Park, Uganda. We monitored trench quality and crop loss to elephants weekly for a year across 47 transects in four park-adjacent communities, and conducted controlled, before-and-after comparisons of verified damage. Elephants damaged or destroyed >4 ha of crops during 48 independent foraging events, the majority <220m from the forest boundary. The frequency of damage varied significantly between and within communities. The majority of trenches were not breached by elephants but five suffered ≥4 breaches. Elephant-breached trenches and their neighboring trenches were lower quality than those not breached in the same week (Wilcoxon test: p<0.001). Trenches were also more likely to be breached where people had planted more crops favored by elephants (Wilcoxon test: p = 0.014). Thus, trench quality and the draw of palatable crops both appeared to influence elephant damage. Although trenches may deter elephants, challenges include heavy labor and difficulties of digging in rocky and swampy areas. Trenches alone will not prevent conflict but this strategy holds promise for hot-spots of conflict at forest edges. Given the stakes for farmers and biodiversity, we call for systematic assessment of crop losses and offer recommendations on monitoring and analysis. Such data will allow for stronger inference about effectiveness before investment of effort and resources in interventions.

## Introduction

Human-wildlife conflict (HWC) around some protected areas threatens local livelihoods and support for conservation. The stakes are particularly high in densely-settled parts of Africa where smallholder farms abut forest parks containing vulnerable species, including elephants (*Loxodonta cyclotis and L*. *africanus*) [1]. Though other wildlife such as baboons (*Papio spp*.) may cause more frequent damage, elephants often have more severe consequences for individual farmers [2–4]. Elephants can destroy entire fields overnight [3], and people lose time, money, and even education or job opportunities if they regularly guard their fields or invest in deterrents [5,6]. Elephants can also be deadly, on occasion seriously injuring or killing people when foraging either in the forest or in adjacent farms.

Conflict perpetuates negative perceptions of protected areas and may spur retaliatory killing of wildlife and initiatives to downsize parks [7,8]. African forest elephants (*L*. *cyclotis*) are critically endangered and most populations have crashed under hunting pressure and habitat loss [9–11], but savannah elephant (*L*. *africanus*) and hybridized African elephant populations are rebounding in some forest parks [12,13]. Rebounding populations confined to parks cannot migrate across their historical range [14], and cause conflict when they cross into agricultural land. Yet to date, at rainforest sites there is limited systematic assessment of elephant crop foraging over time and limited empirical tests of barriers to elephant movement (but see [15], which also highlights how quality of the deterrent or barrier impacts effectiveness).

Efforts to reduce crop damage by elephants often include some type of fence, e.g. beehive [15,16], electric [17] or chili (Capsicum) [18]. These barriers are most successful when deployed at the community level because the large scale of elephant foraging renders individual measures ineffective or may only shift the damage to unprotected farms [3,19,20]. Planting large-scale buffers of low-palatability crops, e.g. tea [21] shows promise but this approach is not feasible for smallholders [22,23]. Sustainability and scalability are key criteria, as strategies that take too much time or money for upkeep at an effective scale are unlikely to persist [19,24,25].

Large trenches have shown promise at some sites and are generally less expensive in terms of materials. Drawbacks of trenches include high labor, possible collapse in heavy rain, and the risk of trapping animals [26,27]. But informal reports of success in deterring elephants at some sites mean trenches warrant a closer look, particularly in rainforest settings [6,28,29]. More broadly, too often the patterns and severity of HWC are assessed only via interviews with local farmers, a vital but incomplete source of information. Given the high stakes for farmers and biodiversity, we encourage systematic assessment of crop foraging patterns. Such data will allow for stronger inference about effectiveness before investment of effort and resources in interventions. Moreover, greater transparency in methods, including definitions of units of analysis and independent samples will help in two ways: one, it conforms to international scientific standards for robust design; two, it facilitates comparisons between study sites.

This study examines the effectiveness of community-scale trenches constructed along the boundary of Kibale National Park, Uganda, where smallholder farmers have a long-documented history of losing crops to wildlife; Kibale has also recorded a few recent incidents of human deaths or serious injuries by elephants. We analyze trench effectiveness at two scales, at the community level and at the transect level, to test how the quality of the trench relates to elephant damage. Crop damage is notoriously hard to quantify systematically in an unbaised fashion due to its stochastic nature in time and space [30], thus we conduct matched-pair analyses to control for both where and when damage occurs. In one set of analyses (within-transect), we compared a damaged transect to itself in another season when elephants did not damage it. In another set of analyses, we compared a segment of trench that elephants breached to nearby trenches that elephants did not breach in the same week. We test whether the presence of abundant highly palatable crops attracts more frequent elephant foraging and/or if the presence of woody unpalatable crops like tea reduces this frequency. Crop composition deserves attention given that farmers at Kibale, and conservationists working elsewhere [22,31], hope that inedible crops will deter wildlife.

## Methods

### Study site

We conducted this study in communities bordering Kibale National Park, Uganda, a 795-km^2^ remnant of mid-altitude (1110-1590m) rainforest that receives an annual rainfall of 1680 mm in two seasons [32]. Kibale’s diverse wildlife includes species notorious for causing crop damage, namely elephants (likely savannah or hybrid savannah-forest elephants [33]) and olive baboons (*Papio anubis*). We focus here on elephants and elephant-specific mitigation strategies (SI for data on crop loss to baboons).

Human population density around Kibale has increased significantly since the park was established in 1994, doubling between 2000 and 2020 to ~300 persons/km^2^ [34]. Land parcels have diminished in size accordingly [35]. People plant staple crops such as plantains, maize, and cassava as well as non-food, high-value crops such as tea, eucalyptus, and coffee [34,36]. Smallholder farmers typically plant crops biannually, during the two rainy seasons (generally in August and March, but depends on the onset of rain).

The risk of crop loss to wildlife is largely shaped by proximity to the park with 90% of elephant damage reported ≤60m of the park edge in 1992–4 [3] and ≤156 m in 2012 [36]. Wildlife caused an average, verified crop loss of 4–7% per farm each year in this high risk zone, but those visited by elephants lost far more [3]. Long-term observation of elephant tracks suggest elephant populations have quadrupled from the 1990s to 2014 [13], and concern over crop loss by elephants has similarly increased [37]. While a growing elephant population is indicative of successful conservation efforts, conflict at Kibale’s edge threatens the livelihoods of smallholders and incidents of retaliatory killing of elephants by farmers have recently been observed (*pers*. *comm*, K. Milich).

Farmers neighboring Kibale have long guarded their fields from wildlife, a strategy of limited success for deterring elephants [3]. Large-scale trench building on site began in 2017 with technical, logistical, and minor material support covered by the Uganda Wildlife Authority (UWA), non-governmental organizations (NGO) such as Conservation to Coexist, and shared tourism revenue [34,38]. At three study sites, local farmers provided nearly all the labor, at the fourth, outside workers were employed by UWA to do initial labor [34,38,39].

### Data collection: Monitoring damage

**Fig 1:**
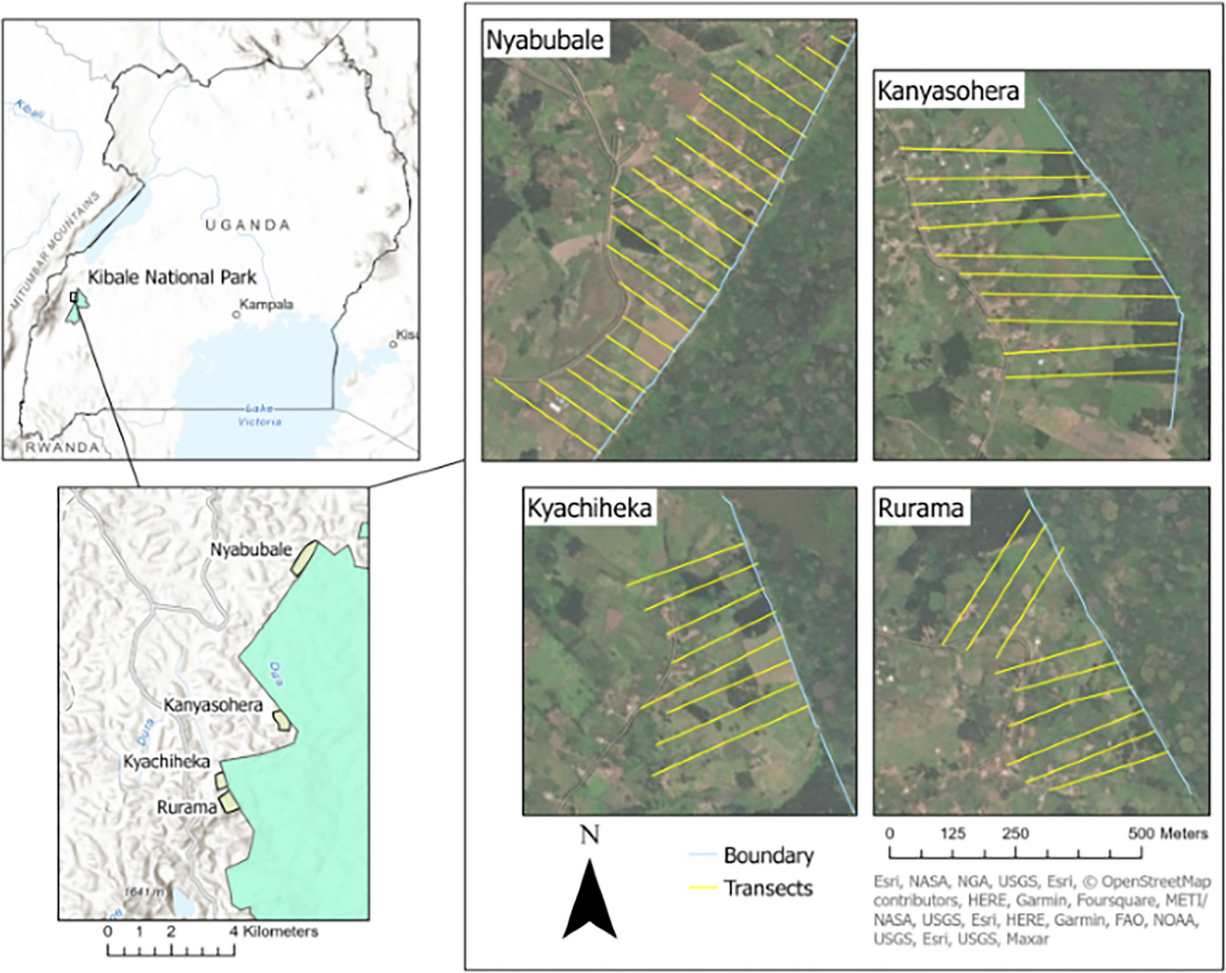
Study communities (n = 4) and transects (n = 47, 50 m wide, 41 to 425 m long (mean = 249 m) bordering Kibale National Park, Uganda.

We collected wildlife crop loss data from August 2020 to August 2021. Every week we surveyed 47 transects running perpendicular to the park edge, each 50 m wide, and extending 41 to 425 meters (mean = 248.8, σ = 83.6) from the park according to presence of crops (as per N-T et al 1998). The 47 transects lie in four communities: Nyabubale, Kanyasohera, Kyachiheka, and Rurama (Fig 1). Each week we canvassed 78 ha total, walking and scanning for wildlife damage in an area covering 223 farm parcels. Small, informal group conversations in May 2022 (one at each of the four study sites) were conducted to check and validate the interpretation of our results, and to discuss challenges of trenches.

At the start of each of two planting seasons, we mapped all crops in every transect at 10 x 25 m resolution. We also mapped eucalyptus, tea, and other non-food, woody plants. Thereafter we canvassed transects weekly, recording all instances of damage by wildlife and estimated number of days since damage occurred. Signs of damage to crops included (but are not limited to) bite marks on fruits or leaves, stalks being broken or torn, or crops pulled from the ground. We identified wildlife responsible based on tracks, dung, bite marks on remaining crops, and any other physical evidence (as per [40]). We measured the damaged crops and measured the extent in square meters. We used a Garmin GPSMAP 64S to record GPS coordinates for each damage point and mapped them in ArcGIS Pro 2.8.0. Last, for every parcel (n = 223) along the transects, we traced the boundaries while on foot using the Garmin devices, then digitized and cleaned up the boundaries in ArcGIS Pro. Land parcels are frequently subdivided, bought and sold, thus 223 (~0.4 ha each) represents a best estimate of the number of independently managed parcels during the study. Precise measurement of parcel size is difficult due to overlapping claims and complex tenurial arrangements. See S1 Table for data on parcel size and S2 Table for non-elephant wildlife damage.

### Data collection: Ranking trench quality

All four communities dug trenches (~3 m deep and 2–3 m wide) for at least some length of the Kibale boundary (range: 350 to 950 m length). Trench quality varied in time and space. Some areas were too swampy or rocky for trenches. We recorded trench quality every week where the transect met the park boundary; hereafter a transect’s trench quality refers to the quality of the trench at the location where the transect line and trench intersect. We used a scale of 0 to 5 where 0 indicates no trench and 5 indicates the trench is ≥3 m deep, ≥2.5 m wide and free of landslips or large boulders. See S1 Protocol for rubric with photo examples.

### Defining independent damage events

To compare crop loss between sites requires transparent definitions of units of analysis and independent samples. If an elephant moves through more than one farm during a single foray outside the park, it could be tallied as one or two events depending on whether one takes the perspective of the elephant or the farmers. Because this study focused on elephant behavior, the appropriate unit of analysis was the foray, defined as the exit from and return to the forest [3]. We define a ‘damage point’ as a set of contiguous crop plants damaged by an animal(s) in a single foray. To focus on elephant behavior and frequency of foraging, we pooled non-independent damage points likely belonging to one foray into a single ‘damage event’. Thus we could analyze statistically independent frequencies of damage events. We determined independence following Naughton-Treves [3]. To pool damage points into the same damage event:

1. Damage points must be caused by the same species
2. Damage points must be recorded in the same week and in the same category of age of events: 0–1, 2–3, or 4–6 days since damage occurred
3. Damage points must have occurred within a given distance, X_v_, of one another point meeting the 1st and 2nd criteria. Points separated by a distance greater than X_v_ reflect damage points are likely caused by independent forays. Naughton-Treves [3] estimated X_v_ for each species separately; for elephants, X_v_ = 710 m.

### Estimating predictor variables

#### Breach points

Because our focus is on whether trenches deter elephants from crossing the park boundary, we sought to estimate where each elephant damage event started (i.e. the trench segment breached). For each independent damage event (as defined above), we defined a ‘breach point’ as the nearest damage point to the park within every damage event; for transects with breach points, we refer to the transects’ trenches having been breached and the transect itself to have been affected. We assumed elephants entered the same breach when foraging as a party and that they breached near the damage site closest to the park, but see below for neighbor trench analysis. Hereafter ‘breach’ refers only to an assumed point at which elephants crossed the park boundary, and ‘damage event’ refers to crop loss in the transect(s) running perpendicular to the trenches.

#### Palatability and buffer crop indices

We defined the *Palability Index* for both transects and for communities as follows. We calculated the number of grid cells (10x25m) planted in a given season with crops that elephants consumed more than five times during the study (banana, bean, cassava, yam, maize, sugarcane, sorghum, and sweet potato). We divided this number by all the total of grid cells in each transects or community. We used this as a proxy for attractiveness to elephants. Also, we calculated a ‘*buffer’ crop index* but replaced the favored crops with woody plants almost never consumed by elephants (eucalyptus, tea, coffee, Misopsis *spp*., and Greveria *spp*.) [3].

#### Trench quality

For community-level analyses, we averaged the trench quality at each transect in each of the four communities over the 52-week study. For transect-level analyses, we used trench quality at the time of the damage. We analyzed three trench quality estimates for each breach: 1) the trench quality for the transect where the breach point was located, 2) the average trench quality for the transect where the breach point was located and the trench quality of its two nearest neighboring transects (one neighbor), and 3) the average trench quality of the transect where the breach point was located and the trench quality of its four nearest neighboring transects (two neighbor). Breach location was estimated, so neighbor analysis allowed for consideration of weak trenches near the breached trench. For transects at the far edge of each community that lacked a neighboring transect, we assessed trench quality once at locations 50 meters and 100 m from the end transect. To do so, we walked along the park boundary until we were 50 m and 100 m from the end transect and recorded the quality of the trench. We observed very little variation in week-to-week trench quality for each transect (average σ **=** 0.36), so we assumed the one-time ranked estimates of quality were sufficiently precise for neighboring trench quality measures (noting that any imprecision could go in either direction).

### Assessing variation in damage between communities, between transects, and within transects over time

We conducted analyses in R with significance set at p ≤ 0.05. To compare the frequency of elephant damage events per transect between communities over the course of the 52-week study period (n = 4 communities), we conducted a non-parametric comparison of summed ranks (Kruskal-Wallis) and report the chi-squared statistic. To test relationships between trench quality, palatability index, or buffer crop index and the frequency of damage events in each community and season, we performed Spearman rank correlations (n = 8 community-season values). At the transect level, we matched affected transects (those with a damage event) to unaffected transects (where no elephant damage event or breach occurred) selected in two ways. In the between-transects comparison, we compared the affected transect to the median trench quality (or median buffer crop index or median palatability index) of all unaffected transects in that same community and same week. We repeated that process but matched affected transects to the same transects in a different season in which it had not been damaged by elephants (hereafter referred to as the within-transects comparison). This method controlled more stringently for variation between transects (within communities) and variation within transects (between seasons).

We analyzed the three trench quality measures for each comparison, reasoning that a transect might be damaged if a neighboring trench were breached or even a trench two neighboring transects away were breached. We also reasoned that the average of affected transects rather than the median would reveal if extremely low- or high-quality trenches affected vulnerability of crops nearby. Our within-transect comparison aimed to understand the influence of other predictors when location was held constant. We used non-parametric tests and paired samples Wilcoxon tests to compare predictors because our indices were ranked.

## Results

### Mapping crops and trench quality

Farmers commonly planted >20 crops including inedible cash crops (‘buffer crops’ and food crops of varying appeal to elephants (Table 1). The average palatability index per transect was 0.36 (σ = 0.18) and the average buffer crop index per transect was 0.32 (σ = 0.22). The most commonly planted buffer crops were eucalyptus and tea, making up about 16% and 10% of transects respectively. Trench quality also varied widely across the communities (e.g. Nyabubale median = 4.1 with the highest score, and Kanyasohera median = 2.5 with the lowest score).

**Table 1:** Characteristics for the four study communities listed from north to south along the border of Kibale National Park, 2020–2021.

Community	Palatability index^a^ Mean	Buffer crop index^b^ Mean	Parcel size Mean ± σ (ha)	Trench quality Mean ± σ
Nyabubale	0.34	0.22	0.60 ± 0.75	4.10 + 1.0
Kanyasohera	0.32	0.49	0.25 ± 0.35	2.48 ± 1.97
Kyachiheka	0.40	0.34	0.92 ± 1.10	4.21 ± 0.58
Rurama	0.54	0.25	0.37 ± 0.83	3.24 ± 1.86
**Mean ± σ**	**0.40**	**0.32**	**0.41 ± 0.69**	**3.61 ± 1.50**

### Patterns of elephant damage

**Fig 2:**
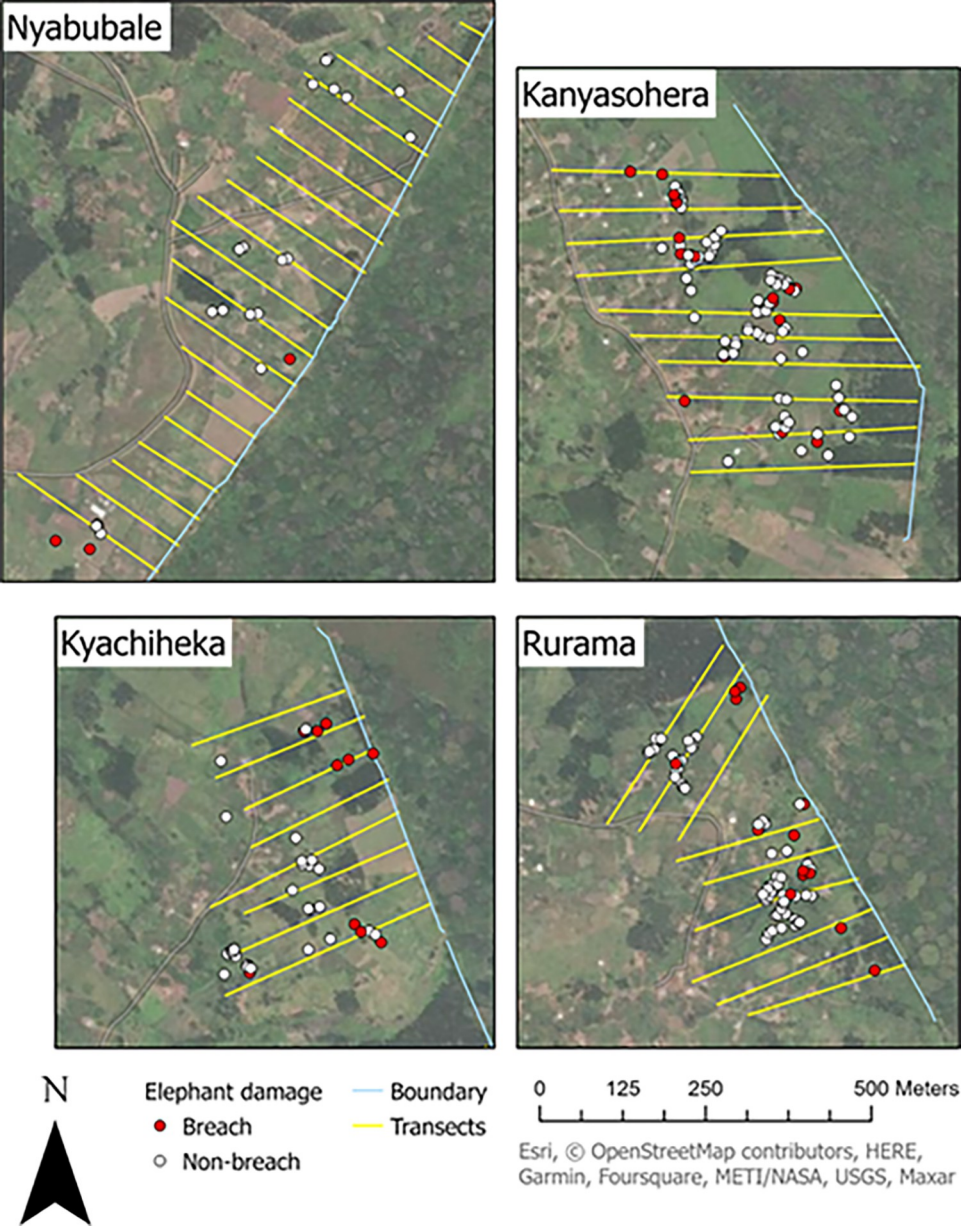
Damage points by community. Elephant damage points in each community (red = breach points (n = 48), white = all other points (n = 178)).

We recorded 226 elephant damage points over 48 independent damage events averaging 145 m from the park boundary and 90% occurred ≤220 m (Fig 2 for map of damage points; S1 Fig for damage distance histogram). Temporally, damage events were distributed equally over the two planting seasons and 52 weeks: there were 28 damage events in season 1, and 20 damage events in season 2 which was slightly shorter due to late rains delaying planting. Damage events averaged about one event per week, with a slight increase at the end of seasons (presumably near harvest time). Of 223 mapped land parcels, elephants damaged 68 (30.5%). The average parcel experienced 1.15 elephant damage points (σ = 3.06, minimum = 0 (155 parcels), maximum = 25 (1 parcel)). Elephants damaged an average of 852 m^2^ per independent damage event (σ = 1085 m^2^) and a maximum of 4,966 m^2^ of damage, mostly affecting a maize field.

**Fig 3:**
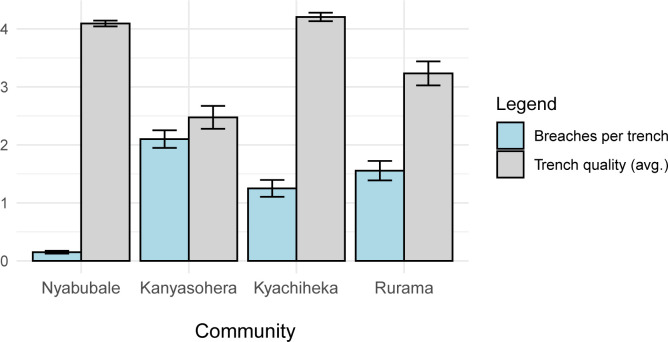
Independent elephant damage events and trench quality (average, with standard deviation) for each community. Communities listed from north to south along Kibale border.

Communities experienced significantly different frequencies of damage events by elephants (Kruskal-Wallis: df = 3, n = 4 X^2^ = 38.4, p < 0.01) (Table 2; Fig 3). Nyabubale had significantly lower frequency than expected by chance, whereas Kanyasohera and Rurama experienced significantly more than expected by chance. These analyses compared communities on the basis of damage events per transect, which would control for the four communities differing in size.

**Table 2:** Elephant damage in four communities (listed north to south) over twelve months, 2020–2021.

Community	Points	Events^a^	Events/ transect Mean	Damage area total (ha)	Damage area (percent relative to total)	% of farms damaged (n = 223)
Nyabubale	22	3	0.15	0.2	0.9	31.6 (12/38)
Kanyasohera	91	21	2.10	0.6	2.8	30.5 (32/105)
Kyachiheka	40	10	1.25	1.2	7.0	39.3 (11/28)
Rurama	73	14	1.56	2.1	12.8	25.0 (13/52)
**Total**	**226**	**48**	**1.02**	**4.1**	**5.2**	**30.5** **(68/223)**

^a^ Damage points grouped into statistically independent foraging ‘events’ based on species responsible, days since damage occurred, and distance between points. See Methods.

**Fig 4:**
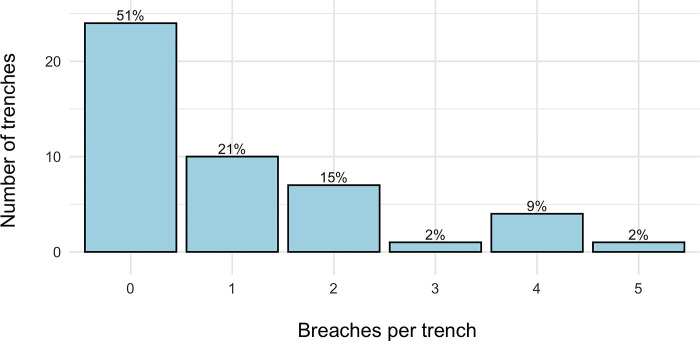
Frequency of independent elephant damage breaches per trench 2020–2021 (trenches n = 47, breaches n = 48).

Five trenches experienced four or more breaches while the majority of trenches (n = 28) experienced zero breaches (total trenches = 47, Fig 4). The five most frequently breached trenches were in Kanyasohera (n = 3) and Rurama (n = 2), which were the two communities with the highest frequency of independent elephant damage events (S2 Fig).

Trench quality, palatability index, and buffer crop index were not significantly related to frequency when independent damage events were split by community-season (S3 Table provides Spearman rank correlation test results). The variability in frequency of damage events and trench quality among the four communities (Fig 3) led us to investigate the variability at the transect level.

### Predicting damage events at the transect level

Spearman tests revealed that the palatability and buffer crop indices were significantly correlated (n = 48, rho = -0.70, p < 0.001). Trench quality was not significantly correlated to the other two predictors (rho = 0.18, p = 0.07 and rho = -0.01, p = 0.93, respectively).

### Trench quality

#### Between-transects

**Fig 5:**
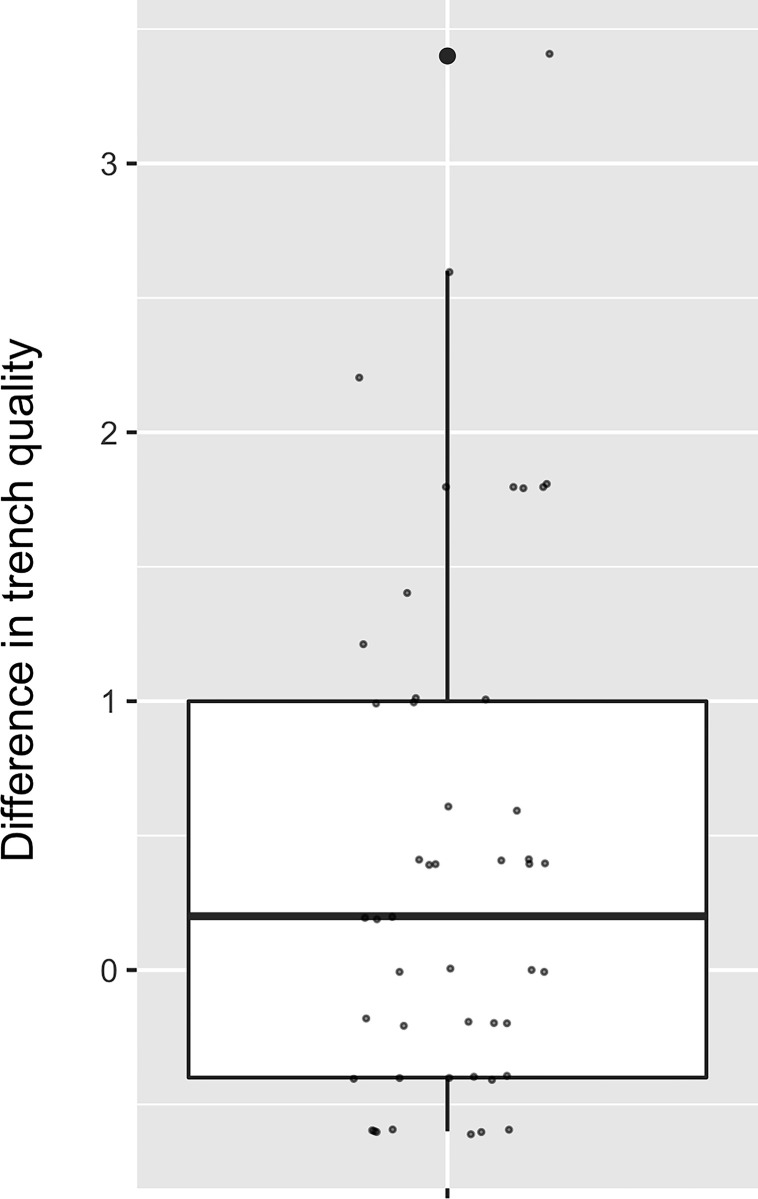
Box plot with points jittered to show difference in “two neighbor” trench quality compared to median (n = 48), (see Methods). A positive value indicates that average quality of affected trench and 4 nearest neighboring trenches were lower quality than median quality of those unaffected in the same community that week. Pairs with differences equal to or less than zero (n = 16, 33%) run counter to these results. Box indicates 25^th^ percentile, median, and 75^th^ percentile; whiskers indicate minimum and maximum values (that are within 1.5*interquartile range, i.e. not outliers).

Affected trenches (breached) were similar in quality to unaffected trenches in the same community in the same week (Wilcoxon test: V = 190.5, p = 0.13). However, taking into account the quality of the “one neighbor” and “two neighbor” trenches, we found breached trenches and their neighbors were significantly lower quality than the median trench quality of unaffected trenches in the same community that week (Wilcoxon test: V = 191, p = 0.001 and V = 201, p < 0.001 respectively) (Fig 5). These results suggest our assumption about location of breach points may be slightly inaccurate because it appears that elephants crossed lower-quality trenches at breach points that were 1–2 transects away from the closest crop damage point.

### Within-transects

Breached trenches had significantly lower quality than the same trench in a random week in the other planting season when it had not been breached (Wilcoxon test: V = 3.5, p = 0.035); one neighbor (Wilcoxon test: V = 10, p = 0.067), and two neighbors (Wilcoxon test: V = 10, p = 0.055).

### Palatability index

#### Between-transects

Transects with elephant damage events had higher palatability indices than the median palatability index of unaffected transects in the same community that week (Wilcoxon test: V = 803.5, p = 0.014) (S3 Fig).

### Within-transects

Transects with damage events did not have significantly higher palatability indices in comparison to the same transect unaffected in the other planting season (Wilcoxon test: V = 564, p = 0.59).

### Buffer crop index

#### Between-transects

Transects with damage events had lower buffer crop indices than the median buffer crop index of unaffected transects in the same community that week (Wilcoxon test: V = 365.5, p = 0.011) (S4 Fig).

### Within-transects

Transects with damage events had similar buffer crop indices as the same transect in a random week of the other season when unaffected (Wilcoxon test: V = 466.5, p = 0.21).

## Discussion

We find evidence of variable elephant damage and elephant breaches, and variable trench quality across transects and communities. Crop damage by elephants remains a concern for all communities but trenches appear to mitigate the damage. Nyabubale, historically the site of highest elephant damage for the same 47 transects [3], during our study recorded the fewest elephant damage points, the fewest breaches, and the smallest total area damaged by elephants. This low loss level is even more striking given that Nyabubale is also the largest study community with 20 transects (~1 km^2^ of park boundary), and the other study communities are smaller but experienced greater damage by nearly all metrics. On average, Nyabubale had the highest quality trenches.

Across the four communities, a majority of transects (51%) had trenches that were not breached, but five transects had trenches that suffered ≥ 4 breaches. This result underscores the patchy distribution of elephant damage at Kibale and elsewhere. We found transects with breached trenches had lower trench quality in their immediate area and when averaged across neighboring trenches than did transects that did not have trenches breached in that same week, and that trenches were in worse shape when breached than in other weeks. Thus we conclude that trenches experiencing more breaches are lower quality than other trenches in the same community, and that trenches may be opportunistically breached when their quality is temporarily poor. We also found that transects with breached trenches had higher crop palatability indices and lower buffer crop indices than other transects at the same time. Since we focus on elephant breaches, as estimated by weekly transect surveys, we may not capture the full picture of elephant crop foraging. Crop loss by wildlife is notoriously difficult to monitor because it is clustered in time and unpredictably located [30]. Elephant crossing points may lie between the transects in our study, e.g. in tea plantations. Palatability indices may need fine-tuning to give extra weight to highly preferred crops. Nonetheless, by locating and monitoring elephant crossing points we revealed that better maintained trenches are less likely to be breached. We also show that trenches do not inevitably collapse in high rainfall sites, a problem noted at other tropical forest sites (Varma et al. 2009).

In May 2022, authors AR, RK and MN shared results via informal conversations at the four sites. Residents generally agreed that well-constructed trenches deter elephants. Some mentioned that land values increased where trenches were well-maintained and elephant forays less frequent. But residents also complained about the high labor of trenches. In three sites, the Uganda Wildlife Authority (UWA) disbursed tourism revenue to the communities to pay their members for digging trenches, and at the fourth, UWA paid outside laborers. The commitment to post-construction maintenance varied between sites and COVID interfered with group maintenance work. At all sites, community members expressed hope for more material and financial support from UWA and NGOs to defray labor costs, and to support beehive fence construction where the park boundary is too rocky or waterlogged for trenches. Last, with regard to woody buffer crops, residents shared that elephants move easily through eucalyptus but do not forage on the trees. Apparently non-palatable crops are a good option for those who can afford it, but smallholders struggling with wildlife damage at the park edge said that they continue planting food crops like sweet potato and cassava (which they note are the worst for wildlife damage) because they do not have the money or food required to try something else or to stop farming.

Beyond testing trenches, >25 years of wildlife damage research at Kibale allows insight on trends. Elephant damage is apparently becoming more frequent [34]. We recorded nearly three times the frequency of elephant foraging events per year as in the same communities in 1998 [3]. The area of crop damage per elephant foraging event appears relatively consistent over time, x = 852, SD = 1085 (1–4966) m^2^ in this study, vs 874 ± 1530 (9–6510) m^2^ in 1998 (Naughton-Treves 1998). Elephants also appear to be foraging further from the park. We recorded 90% of damage ≤ 220 m from the park edge, vs. ≤ 60m in 1992–4 and ≤ 156 m in 2012 [36], respectively). But conclusions about a change in elephant foray distances are complicated by the fact that non-edible woody crops have become more prominent at the park boundary, and nearly all natural forest has been cleared beyond the park boundary. As in 2015, in 2021–22, we find eucalyptus, tea, coffee, and pine make up about 32% of planting ≤ 300 m of the park boundary [36]. The prominence of less-palatable woody crops is likely due to farmers’ efforts to avoid crop loss to wildlife, and the current profitability of these cash crops [36]. As expressed in informal conversations with landholders and as previously documented, renters and those with the smallest parcels must plant food crops, even in risky areas, while wealthier and/or absentee landowners can afford to invest in cash crops [36]. Buffer crops are often recommended in elephant-foraging risk zones around protected areas [41,42].

With elephant populations increasing and a few recent human deaths and serious injuries by elephants in the Kibale area, it is unclear how long trenches will continue to deter elephants or whether local farmers will be willing to continue investing collective labor in maintenance. There is also a risk that trenches may mitigate elephant damage in one community but exacerbate it elsewhere, in a less protected community [20]. But years after they were constructed, the large trenches at Kibale appear to still be offering some protection. Our data also reveal the relationship between a more palatable landscape and elephant breaches, hence a combined approach of maintaining high quality trenches along farms that prioritize non-food crops may be most effective in deterring elephants, as per others’ recommendations for multi-faceted strategies [43]. Other barrier approaches, such as beehive fences, show promise elsewhere but were not our focus because they are only sparsely used at our sites. Beehive fences may also be more appropriate for swampy and rocky areas and would allow a fully protected boundary by using a combination of trenches and beehive fences. A future study may apply similar methods to ours to explore how fence quality may affect where damage or breaches by elephants occur.

Perceptions of wildlife, and therefore protection of wildlife, hinges partly on damage mitigation [34]. Beyond testing trenches or other barriers, we call for more systematic assessment of wildlife damage in areas of conflict. Given the high stakes for local farmers and for threatened wildlife, measuring damage, though time-consuming, is as important as interviews on experience and perception of wildlife encounters or damage by wildlife. Such assessments are all the more important given the wide disparity between risk perception and actual damage. For example, one study revealed that perceived conflict with elephants is as much as seven times greater than independently observed conflict [44]. Measuring damage can help better identify who is most at risk and to prioritize site-specific and species-specific interventions. Efforts to measure and reduce crop damage should focus on elephants and baboons for their potential of severe and frequent damage, respectively.

## Supporting information

S1 Fig Elephant damage distance. Frequency histogram of distance between Kibale National Park boundary and closest edge of a crop damage point by elephants (average distance = 145 m, σ = 64.6 m; 90% of crop damage points fall within 220 m).
(TIFF)

S2 Fig Breaches per trench by community. Frequency of breaches per trench in each community (n = 4) over twelve months of monitoring in 2020–2021 (trenches n = 47, breaches = 48).
(EPS)

S3 Fig Palatability index distribution. Distribution of palatability index for transects with breached trenches and median palatability index of unaffected transects in the same community and week as the transect with the breach. Transects with breached trenches had higher palatability indices than the median palatability index of unaffected transects in the same community that week (Wilcoxon signed rank test, p = 0.014, V = 803.5).
(TIFF)

S4 Fig Buffer index distribution. Distribution of buffer crop index for transects with breached trenches and median buffer crop index of unaffected transects in the same community and week as the transect with the breach. Transects with breached trenches had lower buffer crop indices than the median buffer crop index of unaffected transects in the same community that week (Wilcoxon signed rank test, p = 0.011, V = 365.5).
(TIFF)

S1 Table Parcel results: Number of parcels and average parcel size over time in the Kibale study communities (1992–2021). Parcel size varies and has changed over time. Parcel attributes also differ by community. Tenants renting their land had smaller parcels (average = 0.13 ha, σ = 0.10 ha).
(PDF)

S2 Table All wildlife damage: Summary of crop damage by wildlife in four communities over two planting seasons and twelve months of monitoring (2020–2021). Over 2,444 transect-weeks of data collection resulted in 2,953 records of damage, non-damage, and evidence of guarding or hunting. Overall, we recorded 754 damage points and we pooled these into 278 independent damage events. Baboons caused damage most frequently (480 points in 192 events) but elephants caused more damage in total. Unspecified monkeys (likely cercopithecines), chimpanzees, bushbuck, and birds also accounted for a small amount of damage. Together, all wildlife species caused 6.6 ha of damage during the entire study period, which is 8.5% of the study area.
(PDF)

S3 Table Spearman rank correlation test results. Spearman rank correlation test results testing relationships of average trench quality, palatability index, and buffer crop index against breaches/transect.
(PDF)

S1 Protocol Trench rubric. (PDF)

S1 Dataset Raw data files of crop monitoring and crop mapping data. (ZIP)

S1 File R code and related.csv files used for analysis. (ZIP)
