# Supplementary figures and images for: Trenches reduce crop foraging by elephants: Lessons from Kibale National Park, Uganda for elephant conservation in densely settled rural landscapes
Source: PLoS One. 2023 Jul 26;18(7):e0288115. doi: 10.1371/journal.pone.0288115 (PMC10370685; doi:10.1371/journal.pone.0288115)

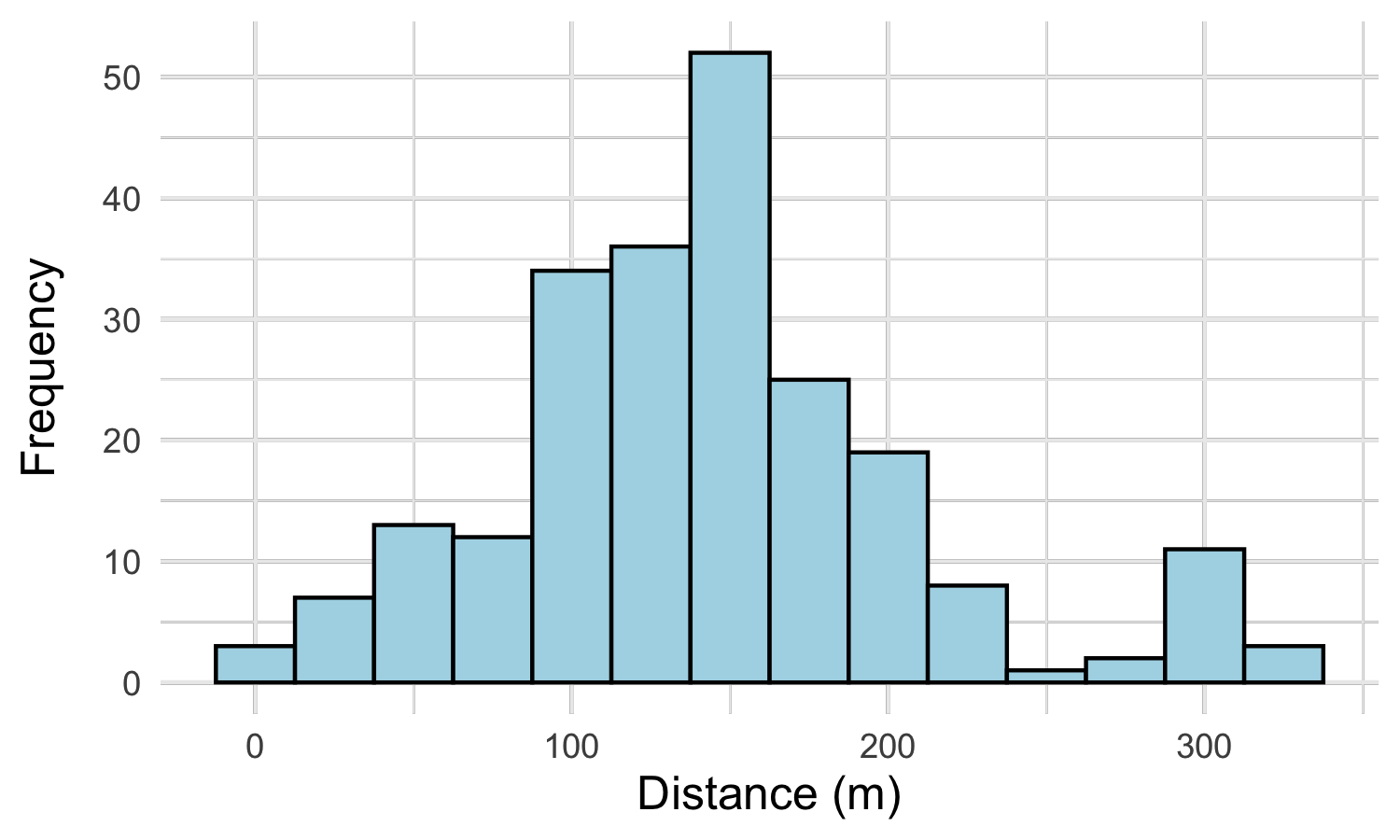

Supplement: S1 Fig — Frequency histogram of distance between Kibale National Park boundary and closest edge of a crop damage point by elephants (average distance = 145 m, σ = 64.6 m; 90% of crop damage points fall within 220 m). (TIFF) [file pone.0288115.s001.tiff]

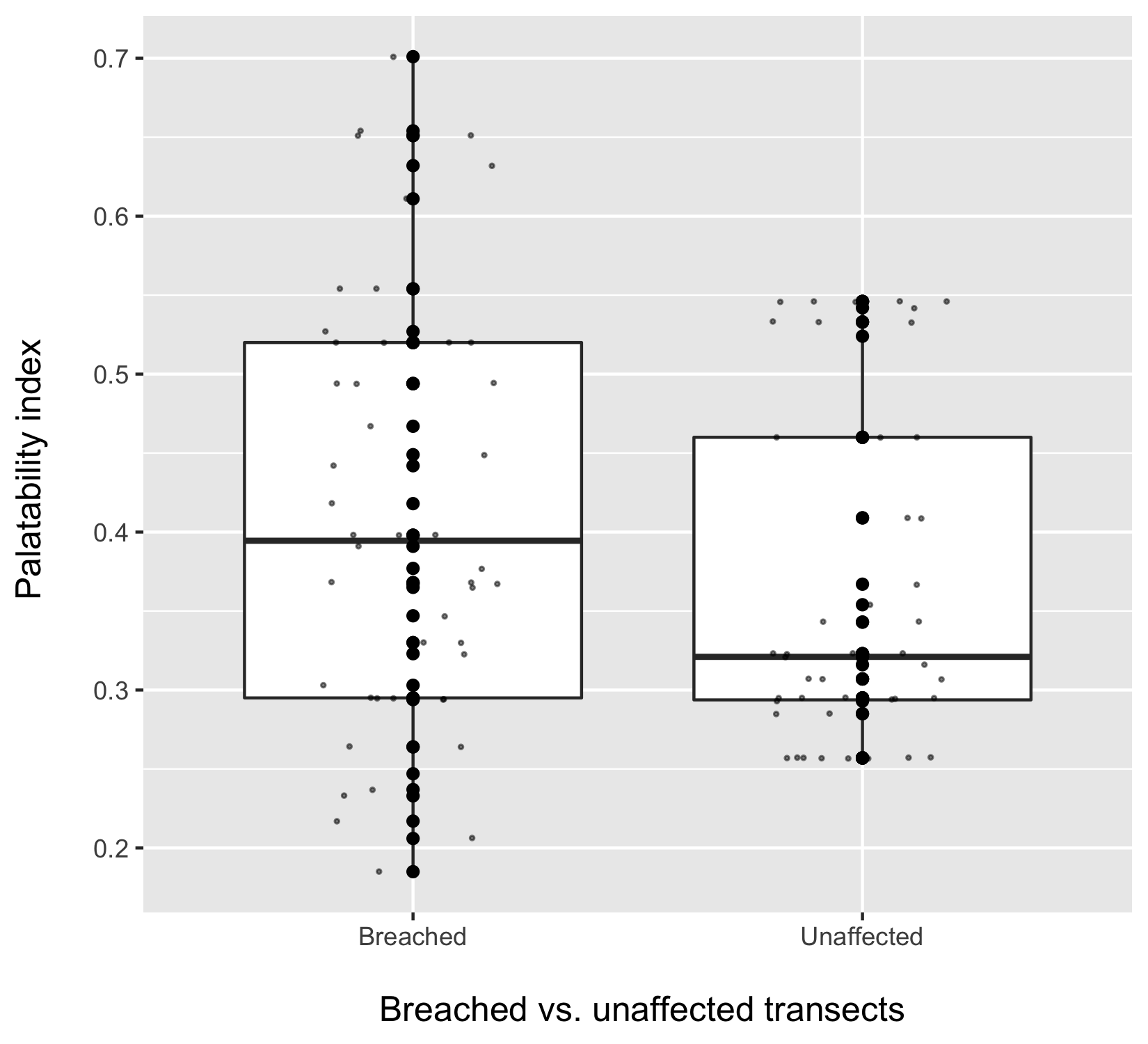

Supplement: S3 Fig — Distribution of palatability index for transects with breached trenches and median palatability index of unaffected transects in the same community and week as the transect with the breach. Transects with breached trenches had higher palatability indices than the median palatability index of unaffected transects in the same community that week (Wilcoxon signed rank test, p = 0.014, V = 803.5). (TIFF) [file pone.0288115.s003.tiff]

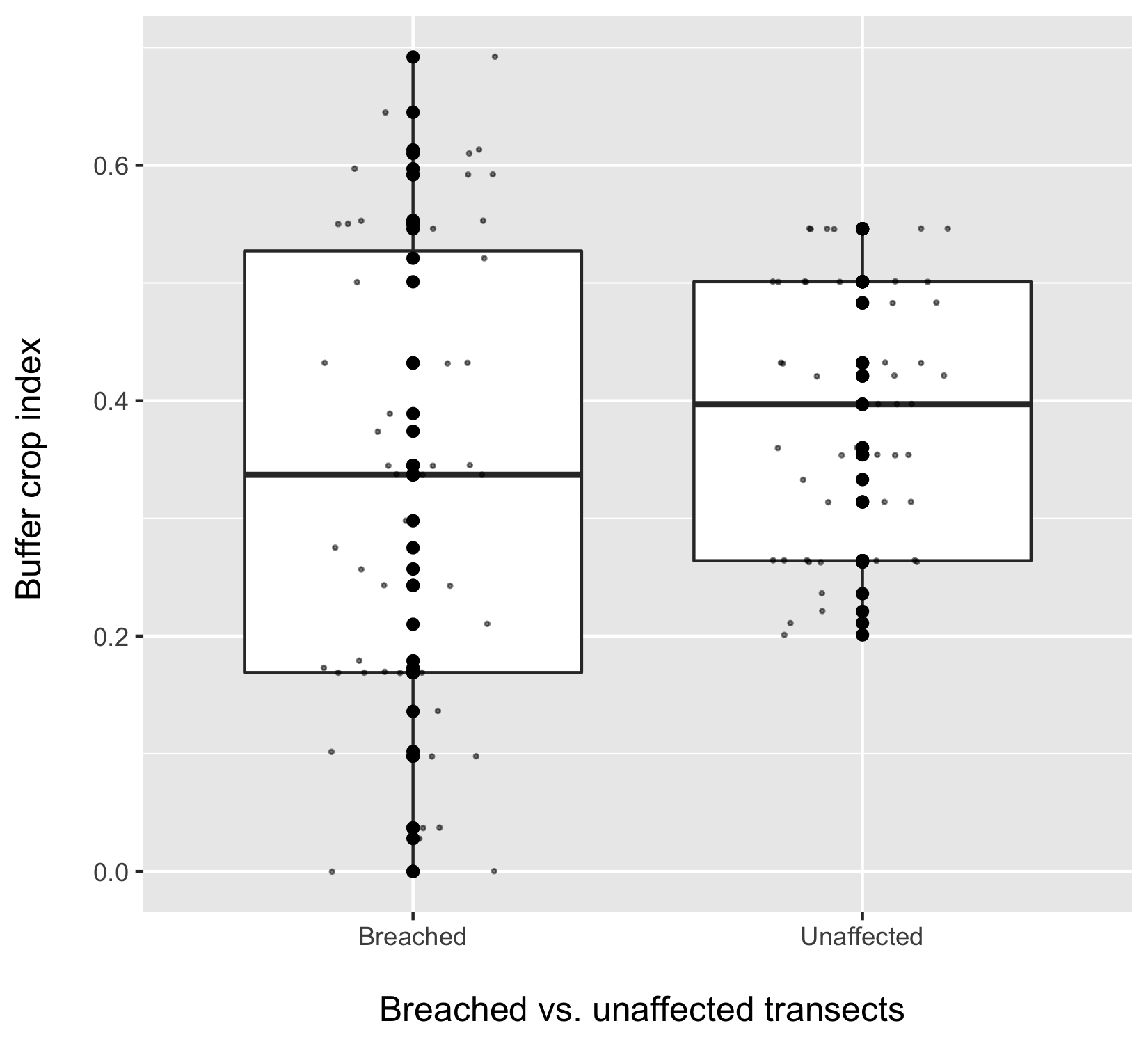

Supplement: S4 Fig — Distribution of buffer crop index for transects with breached trenches and median buffer crop index of unaffected transects in the same community and week as the transect with the breach. Transects with breached trenches had lower buffer crop indices than the median buffer crop index of unaffected transects in the same community that week (Wilcoxon signed rank test, p = 0.011, V = 365.5). (TIFF) [file pone.0288115.s004.tiff]
