# Supplementary material for: Trenches reduce crop foraging by elephants: Lessons from Kibale National Park, Uganda for elephant conservation in densely settled rural landscapes
Source: PLoS One. 2023 Jul 26;18(7):e0288115. doi: 10.1371/journal.pone.0288115 (PMC10370685; doi:10.1371/journal.pone.0288115)
Supplement: S1 Table — Parcel size varies and has changed over time. Parcel attributes also differ by community. Tenants renting their land had smaller parcels (average = 0.13 ha, σ = 0.10 ha). (PDF) [file pone.0288115.s005.pdf]

S1 Table. Parcel Results

Parcel size varies and has changed over time. Parcel attributes also differ by community. Tenants renting their land had smaller parcels (average = 0.13 ha, σ = 0.10 ha).

Number of parcels and average parcel size over time in the Kibale study communities (1992 - 2021)[a]

**2020-2021**		**1992-1994**[b]		**2015**[c]	
Number of parcels	Parcel size Mean ± σ (Range) (ha)	Number of parcels	Parcel size Mean (ha)	Number of landusers *	Parcel size Mean (ha)
223	0.37 ± 0.67 (0.01 - 4.40)	97	1.4	153	1.2

[a] Note study sites overlap but are not identical due to removing Kabucikire from this study.

[b] From Naughton-Treves, 1997.

[c] From L'Roe & Naughton Treves, 2017. *Number of parcels was not reported. Note average parcel size for renters is also reported: 0.4 ha.
