# Supplementary material for: Trenches reduce crop foraging by elephants: Lessons from Kibale National Park, Uganda for elephant conservation in densely settled rural landscapes
Source: PLoS One. 2023 Jul 26;18(7):e0288115. doi: 10.1371/journal.pone.0288115 (PMC10370685; doi:10.1371/journal.pone.0288115)
Supplement: S2 Table — Over 2,444 transect-weeks of data collection resulted in 2,953 records of damage, non-damage, and evidence of guarding or hunting. Overall, we recorded 754 damage points and we pooled these into 278 independent damage events. Baboons caused damage most frequently (480 points in 192 events) but elephants caused more damage in total. Unspecified monkeys (likely cercopithecines), chimpanzees, bushbuck, and birds also accounted for a small amount of damage. Together, all wildlife species caused 6.6 ha of damage during the entire study period, which is 8.5% of the study area. (PDF) [file pone.0288115.s006.pdf]

S2 Table. All Wildlife Damage

Over 2,444 transect-weeks of data collection resulted in 2,953 records of damage, non-damage, and evidence of guarding or hunting. Overall, we recorded 754 damage points and we pooled these into 278 independent damage events. Baboons caused damage most frequently (480 points in 192 events) but elephants caused more damage in total. Unspecified monkeys (likely cercopithecines), chimpanzees, bushbuck, and birds also accounted for a small amount of damage. Together, all wildlife species caused 6.6 ha of damage during the entire study period, which is 8.5% of the study area.

Summary of crop damage by wildlife in four communities over two planting seasons and twelve months of monitoring (2020-2021).

**Species**	**Points**	**Events** [a]	**Events/ transect Mean**	**Damage per event Mean ± σ (range) (m^2)**	**Damage area total (ha)**	**Damage area (percent relative to total farm area)**	**% of farms damaged (n = 223)**
Elephants	226	48	1.02	851 ± 1085 (1 - 4966)	4.09	5.23	30.5% (68/223)
Baboons	480	192	4.09	134 ± 195 (0.3 - 1406)	2.35	3.01	36.3% (81/223)
Other wildlife (monkeys, birds, chimpanzees) [b]	48	38	0.81	64 ± 95 (0.25 - 400)	0.17	0.22	6.7% (15/223)
**Total**	**754**	**278**	**5.92**		**6.62**	**8.5% (6.6/78.2)**	**45.3% (101/223)**

[a] Grouped nonindependent damage points into statistically independent foraging 'events' based on species responsible, days since damage occurred, and distance between points.

[b] Monkeys (n = 42), birds (n = 3), and chimpanzees (n = 3). Bushbuck were also observed (n = 3) in Kabucikire, not included in any analyses.
