# Supplementary material for: Trenches reduce crop foraging by elephants: Lessons from Kibale National Park, Uganda for elephant conservation in densely settled rural landscapes
Source: PLoS One. 2023 Jul 26;18(7):e0288115. doi: 10.1371/journal.pone.0288115 (PMC10370685; doi:10.1371/journal.pone.0288115)
Supplement: S3 Table — Spearman rank correlation test results testing relationships of average trench quality, palatability index, and buffer crop index against breaches/transect. (PDF) [file pone.0288115.s007.pdf]

S6 Table. Spearman Rank Correlation Test Results

Table S6. Spearman rank correlation test results testing relationships of average trench quality, palatability index, and buffer crop index against breaches/transect.

**Variable**	**by Variable**	**Spearman ρ**	**Prob>|ρ|**
Avg_trench	breach/transect	-0.5714	0.1390
palatability	breach/transect	-0.3234	0.4346
palatability	Avg_trench	0.2275	0.5878
buffer	breach/transect	0.7066	0.0501
buffer	Avg_trench	-0.5868	0.1262
buffer	palatability	-0.6145	0.1050
