# Supplementary material for: Trenches reduce crop foraging by elephants: Lessons from Kibale National Park, Uganda for elephant conservation in densely settled rural landscapes
Source: PLoS One. 2023 Jul 26;18(7):e0288115. doi: 10.1371/journal.pone.0288115 (PMC10370685; doi:10.1371/journal.pone.0288115)

S3 Protocol. Trench Rubric

TRENCH AND HIVE QUALITY MONITORING PROTOCOL

1. Quality of trenches and beehive fences will be monitored and recorded weekly for each site (at time of crop raiding monitoring). Field assistants will work together to determine quality of the trench or beehive fence on a scale of 1 (bad) to 5 (excellent) along the park boundary.
2. The goal is to map the trench or beehive quality over time along the park border at sites where we are monitoring transects for crop damage.
3. Field assistants will start data collection 100 meters away along the park boundary from the first transect point for the site. You may work in either direction along the park boundary (north to south or south to north)
4. Quality of the trenches and beehive fences are assessed on a scale of 1 to 5

1. Very poor (not at all effective)

Trench or beehive fence is visible but broken, kicked in, and easy for an elephant to pass through. Damage is <u>extreme</u>, and maintenance is not recent. Trench is almost filled in or sides are very sloped from elephant damage. Beehive fence is toppled.

2. Poor (sometimes effective)

Trench or beehive fence is visible and poorly constructed but may slow or prevent elephants, but many elephants are able to pass through. Trench sides show sloping from elephant damage or is partly filled in. Beehive fence is falling down so elephants can step over or push through.

3. Okay (fairly effective)

Trench or beehive fence is present but has some damage. Some elephants are deterred but some can still pass through.

4. Good (very effective)

Trench or beehive fence is constructed and maintained but may have some damage or problem allowing some few elephants to pass through. Trench may have small amounts of soil kicked in.

5. Excellent (extremely effective)

Trench or beehive fence is very well constructed and maintained. Elephants are not able to pass through.

PHOTO KEY:

1 - very poor (very damaged or muddy, easy to cross, has traces of what used to be a trench)

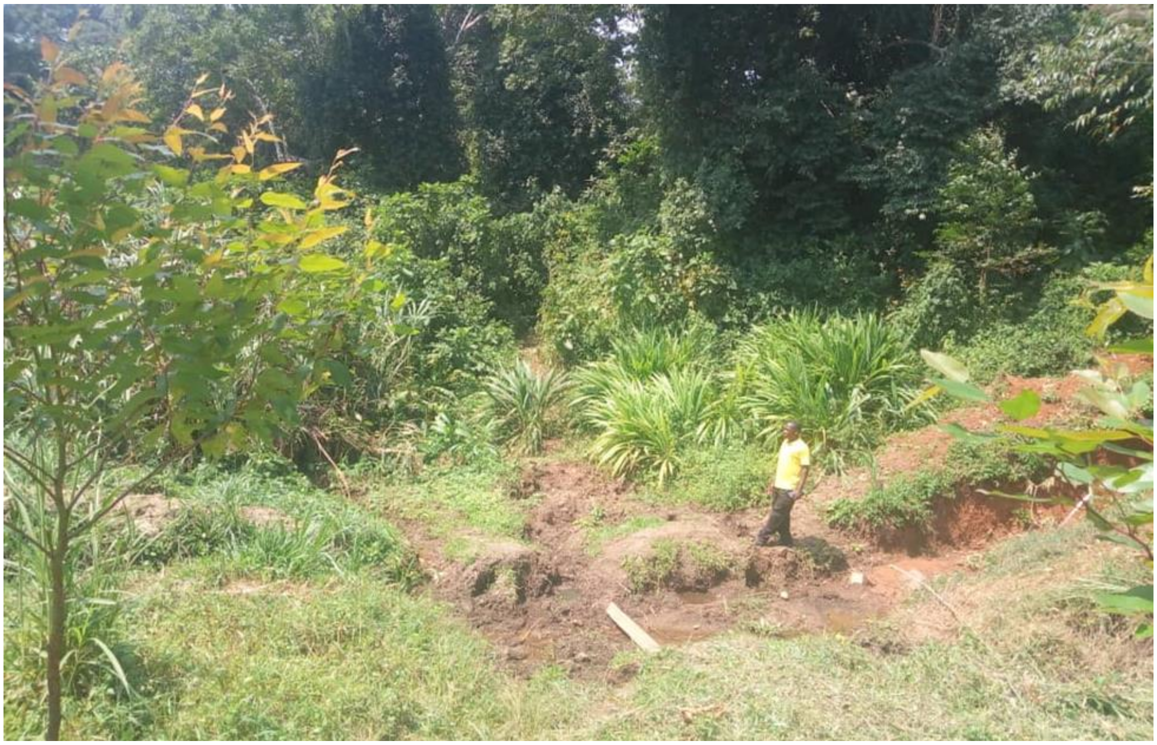

2 - poor (very damaged, easy to cross, slightly more established than [1])

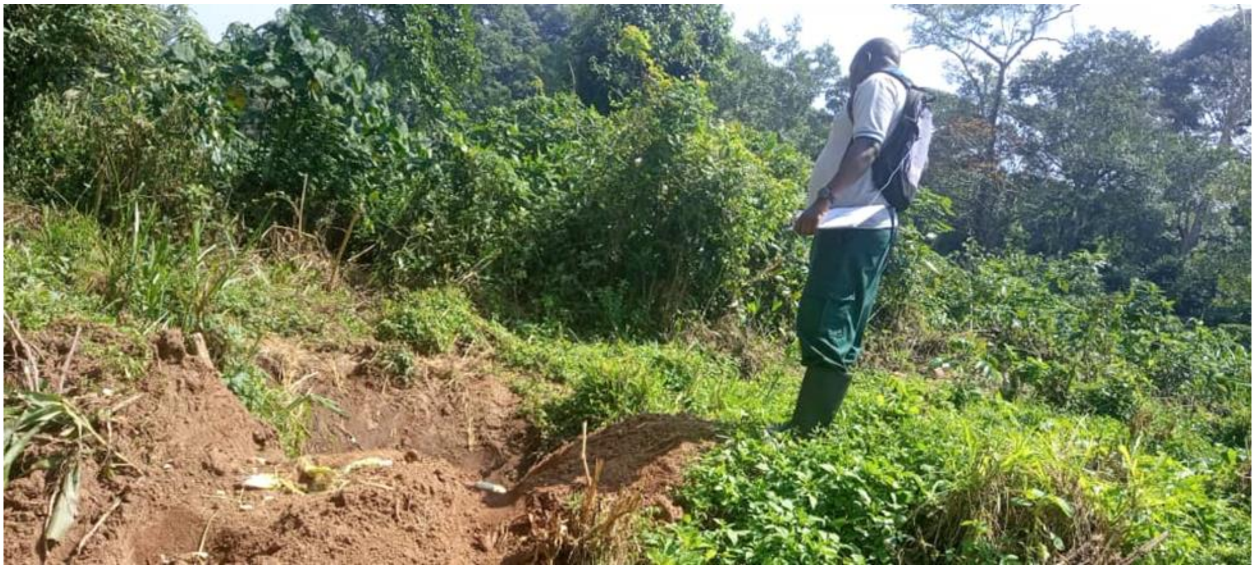

3 - fair (moderate condition, may have rocks or muddy sections, not deep or wide, still crossable by animals, could be fixed to be a 5)

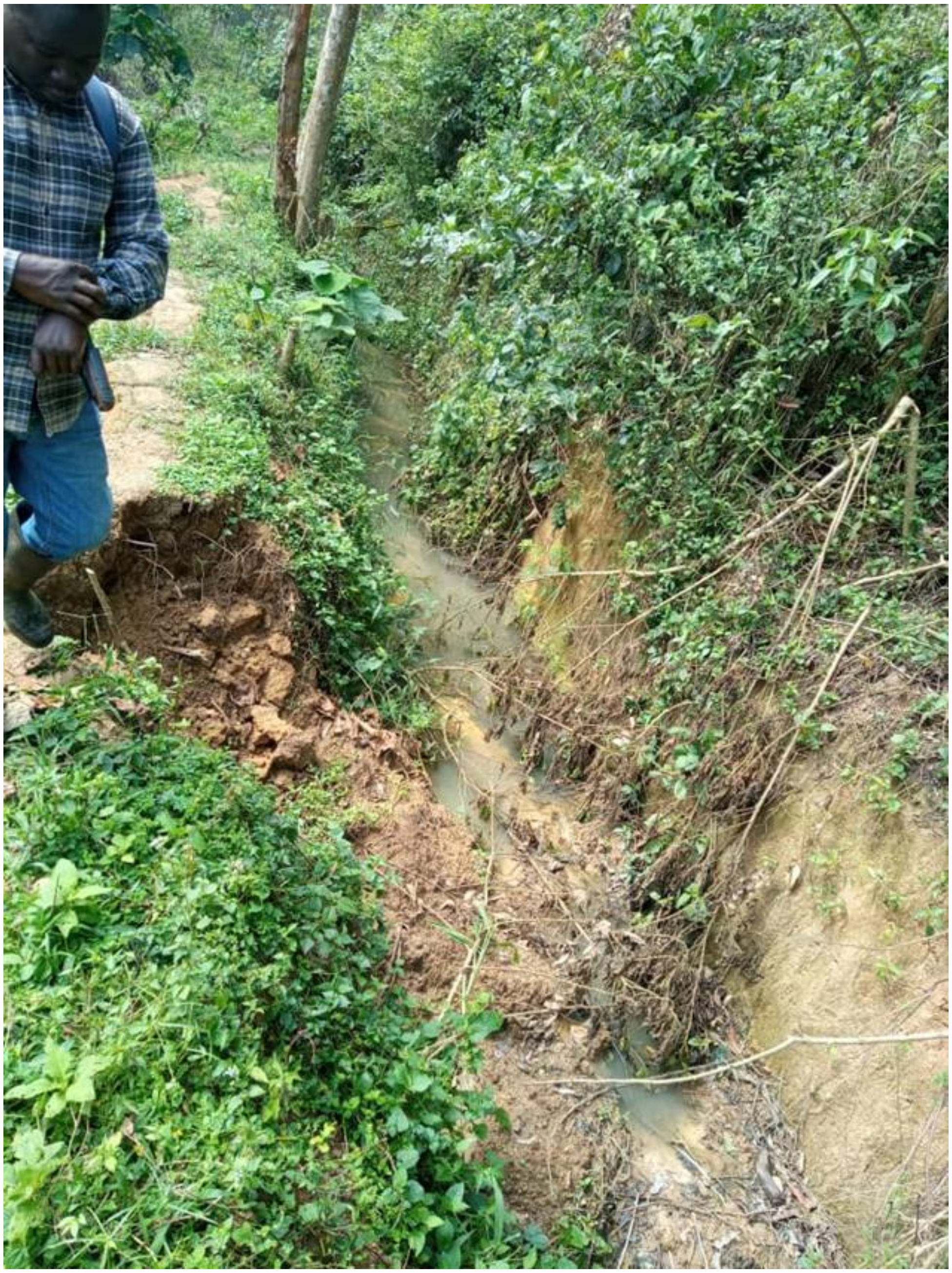

4 - good (well constructed, deep and wide but may have some pushed in soil or other vulnerabilities, could be fixed to a 5)

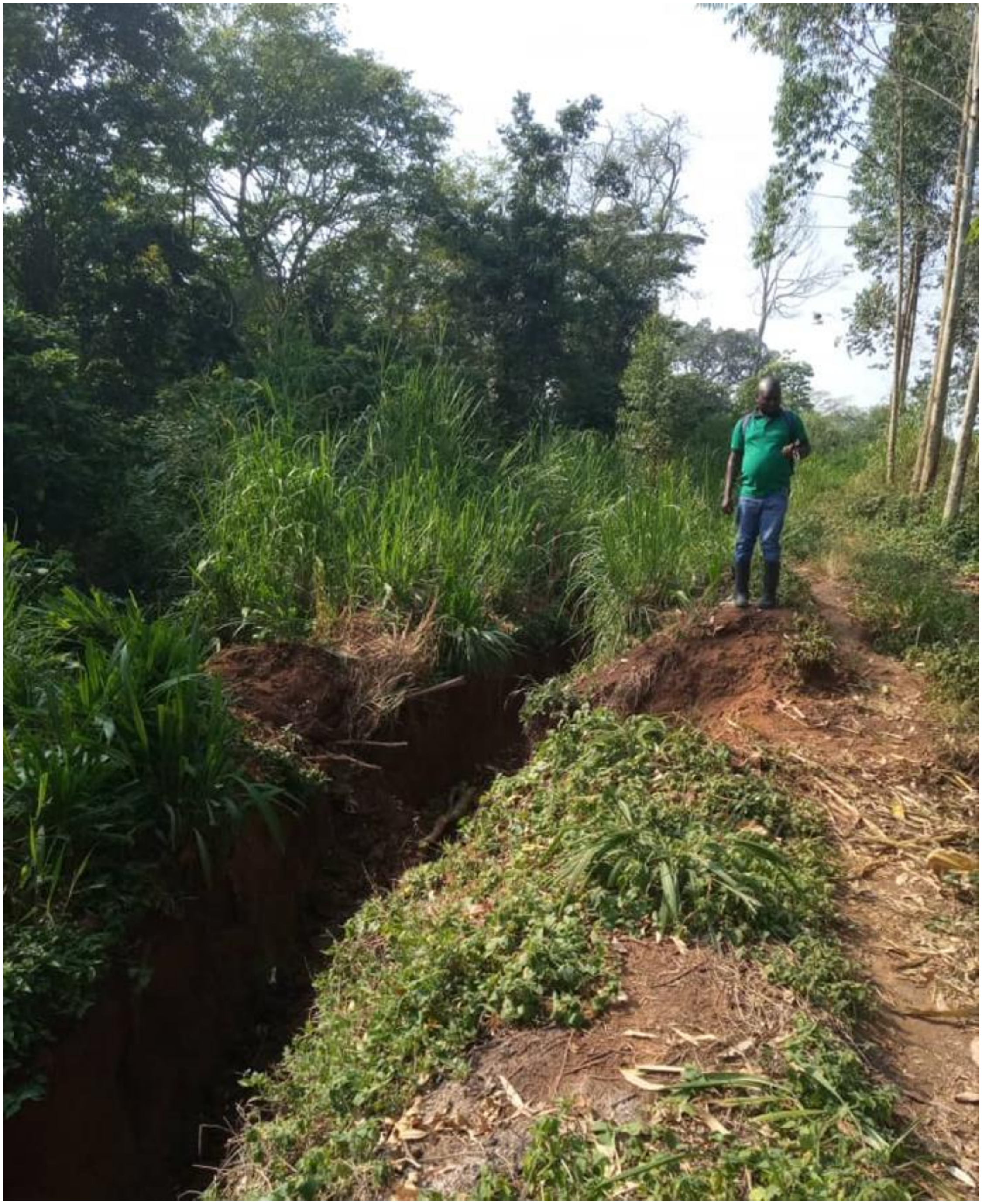

5 - excellent (well constructed, good trench sides, deep and wide, cannot cross, 2-3m wide and 3m deep)

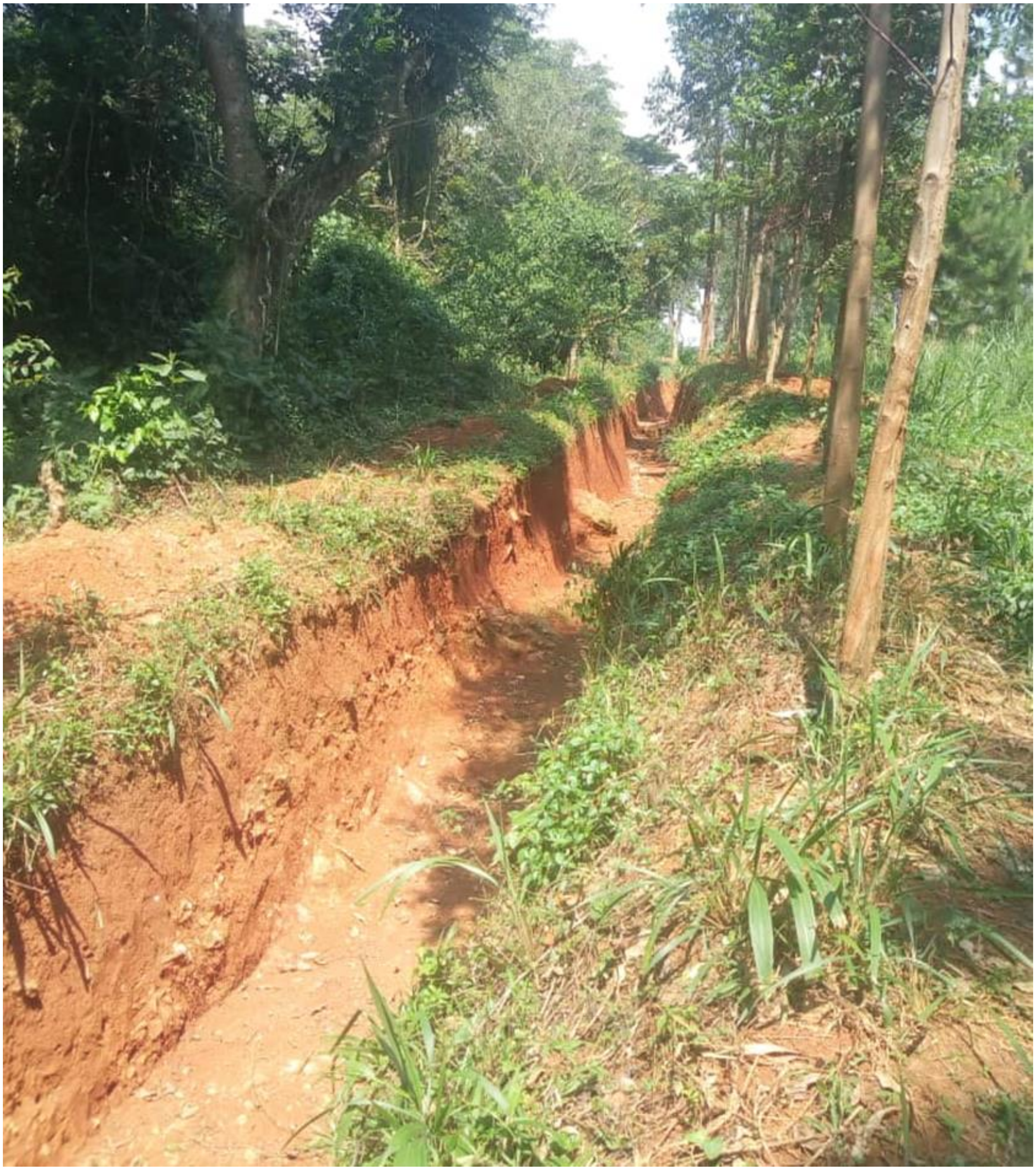

Supplement: S1 Protocol — (PDF) [file pone.0288115.s008.pdf]
